# Low-Temperature Growth of ZnO Nanowires from Gravure-Printed ZnO Nanoparticle Seed Layers for Flexible Piezoelectric Devices

**DOI:** 10.3390/nano11061430

**Published:** 2021-05-28

**Authors:** Andrés Jenaro Lopez Garcia, Giuliano Sico, Maria Montanino, Viktor Defoor, Manojit Pusty, Xavier Mescot, Fausta Loffredo, Fulvia Villani, Giuseppe Nenna, Gustavo Ardila

**Affiliations:** 1 University Grenoble Alpes, University Savoie Mont Blanc, CNRS, Grenoble INP, IMEP-LaHC, F-38000 Grenoble, France; andres-jenaro.lopez-garcia@grenoble-inp.fr (A.J.L.G.); viktor.defoor@grenoble-inp.fr (V.D.); manojit.pusty@grenoble-inp.fr (M.P.); xavier.mescot@grenoble-inp.fr (X.M.); 2 ENEA, Italian National Agency for New Technologies, Energy and Sustainable Economic Development, Portici Research Centre, P.le E. Fermi 1, Portici, I-80055 Naples, Italy; giuliano.sico@enea.it (G.S.); maria.montanino@enea.it (M.M.); fausta.loffredo@enea.it (F.L.); fulvia.villani@enea.it (F.V.)

**Keywords:** piezoelectric sensor, mechanical energy harvesting, nanogenerator chemical synthesis, gravure printing, flexible electronics

## Abstract

Zinc oxide (ZnO) nanowires (NWs) are excellent candidates for the fabrication of energy harvesters, mechanical sensors, and piezotronic and piezophototronic devices. In order to integrate ZnO NWs into flexible devices, low-temperature fabrication methods are required that do not damage the plastic substrate. To date, the deposition of patterned ceramic thin films on flexible substrates is a difficult task to perform under vacuum-free conditions. Printing methods to deposit functional thin films offer many advantages, such as a low cost, low temperature, high throughput, and patterning at the same stage of deposition. Among printing techniques, gravure-based techniques are among the most attractive due to their ability to produce high quality results at high speeds and perform deposition over a large area. In this paper, we explore gravure printing as a cost-effective high-quality method to deposit thin ZnO seed layers on flexible polymer substrates. For the first time, we show that by following a chemical bath deposition (CBD) process, ZnO nanowires may be grown over gravure-printed ZnO nanoparticle seed layers. Piezo-response force microscopy (PFM) reveals the presence of a homogeneous distribution of Zn-polar domains in the NWs, and, by use of the data, the piezoelectric coefficient is estimated to be close to 4 pm/V. The overall results demonstrate that gravure printing is an appropriate method to deposit seed layers at a low temperature and to undertake the direct fabrication of flexible piezoelectric transducers that are based on ZnO nanowires. This work opens the possibility of manufacturing completely vacuum-free solution-based flexible piezoelectric devices.

## 1. Introduction

Flexible piezoelectric transducers make the development of innovative devices possible, especially in the energy and sensor fields [1]. Several piezoelectric materials have been studied and integrated into flexible substrates, including organic materials, such as poly(vinylidene fluoride) (PVDF) [2] and its co-polymers [3,4,5]; inorganic materials, like lead zirconate titanate (PZT) [6], potassium sodium niobate (KNN) [7], lead magnesium niobate-lead titanate (PMN-PT) [8], barium titanate (BaTiO_3_) [9], zinc stannate (ZnSnO_3_) [10], and bismuth ferrite (BiFeO_3_) [11]; and semiconductors like zinc oxide (ZnO) in the shape of nanowires (NWs) [12,13]. Among those materials, ZnO has attracted a great deal of attention because it is biocompatible, abundant, and sustainable, making it interesting for future applications while reducing the use of critical and toxic materials [14]. In particular, ZnO NWs have been widely used in different applications related to the conversion of mechanical energy into electricity at the nanometric scale. Some advantages of these NWs, compared to the bulk counterpart, are the ability to withstand high deformation values without fracturing [15,16,17], high values for the Young’s modulus [18,19], and high values for the piezoelectric coefficient [20], as well as a high surface-to-volume ratio that allows enhanced piezoelectric responses thanks to Fermi-level pinning [21].

ZnO can exist in different miniaturized forms, like quantum dots, nanorods, nanowires, nanotubes, nanosheets, nanobelts, nanocones, and twinned disk structures [22]. The ability to form different shapes in miniature sizes, along with a high surface charge density, polarity, and orientation enables ZnO nanostructures to display various applications in the fields of gas sensing, energy storage, energy harvesting, photocatalysis, flexible electronics, optoelectronics, and environmental waste treatment, etc. The high surface area of ZnO NWs promotes greater interfacial adsorption and diffusion, which enhances their physical and chemical properties. ZnO has a wide band gap of 3.37 eV, which is one of the highest in the II-IV semiconductor metal oxides, and also has a large excitation binding energy of 60 meV [23].

These superior properties make ZnO NWs an interesting material to be adopted in the high-tech renewable energy field for the development of energy conversion or storage devices, as ZnO NWs meet the requirements for power and energy density [24]. Also, the low cost of fabrication, negligible emission of pollutants, and the possibility of enhancing energy conversion efficiency make ZnO NW-based devices economically viable for industrial production [25,26].

The first energy harvesting device based on ZnO NWs (then referred to as a nanogenerator—NG) was demonstrated in 2006 by Wang et al. [27]. Since then, a number of NG devices with different structures that are grown on flexible or rigid substrates have been investigated. Recently, in the field of flexible electronics, a great deal of effort has been focused on flexible NG devices, which can be used while experiencing bending [28,29,30] or compressive [31,32,33,34,35] forces in applications like material-based flexible electronic skins [36] or as nanocomposite-based sensors [37].

Chemical bath deposition (CBD) is a particularly popular method to grow piezoelectric nanostructures due to its ease of implementation, support to scale production, and moderate processing temperatures [33,38,39]. With low processing temperatures, the method is suitable for the use of flexible plastic substrates. Usually, the substrate is covered by a thin seed layer to promote growth and lower the thermodynamic nucleation barrier [40]. Once the seed layer is deposited, the substrates are immersed in a supersaturated growth solution at a moderate temperature for several hours. Using this technique, ZnO NWs or nanorods (NRs) have been grown over metallic [41,42,43] and polymeric materials [30,44,45], depending on the type of ZnO seed layer deposition. In this work, zinc nitrate hexahydrate (Zn(NO_3_)_2_.6H_2_O) and hexamethylenetetramine ((CH_2_)_6_N_4_) are used as precursors for the synthesis of ZnO NWs using the CBD process. Hexamethylenetetramine (HMTA) is a popular reagent that is used as a hydroxide anion (OH^−^) precursor for ZnO synthesis [46].

To date, a large variety of techniques are used to obtain ZnO seed layers, such as RF sputtering, atomic layer deposition (ALD), spray pyrolysis, chemical vapor deposition, and wet chemical synthetic routes, including spin and dip coatings [47,48]; however, most of them are confined to a laboratory scale and require an additional procedure for preparing patterned layers through expensive and complex processes such as photolithography and etching [49]. Moreover, the typical synthetic routes usually require high temperature annealing post-treatment for improving the crystallinity of the ZnO seeds, thus excluding polymers from the choice of flexible substrates [50,51,52].

On the other hand, there has been an increasingly pressing demand for easy and cost-effective methods to prepare ZnO patterned seed layers at low temperatures [49,53], which is essential for the use of plastic substrates.

In the last decade, conventional printing processes have been highly regarded as additive techniques for easy, fast, and low-cost thin film deposition at ambient conditions [54,55]. Using these techniques, patterning is carried out simultaneously with the deposition, thus substantially reducing energy, time, and material consumption [56]. In addition, the typical low temperatures of solution processes make printing compatible with most flexible substrates [55,57]. Among printing techniques, gravure techniques are widely employed in many fields and applications thanks to the ability to couple high throughput and high quality [58,59]. Recently, gravure printing has been proven to support the production of uniform large-scale nanoparticulate ZnO thin films on flexible plastic substrates [60]. The quality of the deposited films is so high that it successfully allows subsequent ZnO sintering through an innovative method at a very low temperature and in pressure less conditions [61]. Several printing techniques have been used to deposit ZnO seed layers for the subsequent growth of ZnO NWs, although none of the reports show piezoelectric measurements with the grown NWs [57]. Furthermore, to the best of our knowledge, there have been no reports on the growth of ZnO NWs from layers deposited by gravure printing.

**Figure 1:**
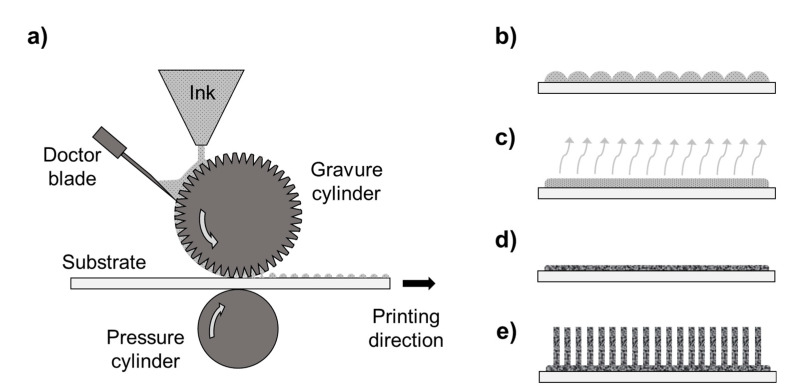
Fabrication process of ZnO seed layers by gravure printing and subsequent NW growth. The gravure printing process can be divided in the following steps: (**a**) inking, doctoring, and transfer; (**b**) spreading; (**c**) drying; (**d**) production of the final solid thin film. (**e**) The final growth of ZnO NWs is performed via low-temperature CBD.

Gravure printing techniques rely on the direct transfer of a low-viscosity ink (1–100 mPas) from the micro-engraved cells of a cylinder to a flexible substrate by the pressure of a counter cylinder. This process can be considered as a sequence of sub-processes (inking, doctoring, transfer, spreading, and drying, as shown in Figure 1a–d) [60]. Essentially, at the microscopic level, the fluid dynamics of the gravure printing process are governed by the balance between viscous and surface tension forces, where the latter are the driving force [62].

In this study, gravure printing with crystalline ZnO nanoparticles (NPs) was attempted for the first time to produce a seed layer for ZnO NW growth (Figure 1e) from the perspective of flexible piezoelectric devices manufacturing. Using crystalline NPs as starting material allows the elimination of the thermal post-annealing step, making the process easier, time-effective, and compatible with polymeric substrates. Two morphologies of the seed layer were also produced (i.e., nanoparticulate and sintered) to investigate their influence on the density, the diameter, and the orientation of NW growth. The NWs grown from the gravure-printed seed layers were also compared with ZnO NWs grown from a seed layer deposited by ALD on a silicon substrate as a reference.

Piezoelectric properties, including the amplitude of the piezo-response and the phase of the grown NWs, were measured with the different samples via piezo-response force microscopy (PFM).

## 2. Experiments

### 2.1. Seed Layer Deposition

A ZnO NP colloidal suspension (Sigma-Aldrich, St. Luis, MO, USA, particle size <130 nm) was used to prepare an ink, diluting the commercial suspension with ethanol (final concentration 8 wt %), for printing seed layers of a 55 ± 6 nm size onto a commercial indium tin oxide (ITO)/poly(ethylene terephthalate) (PET) (150 nm/125 µm) substrate (2 × 1.5 cm^2^) acquired from DuPont Teijin Films, Luxembourg.

Printing was performed using a lab-scale gravure printer (IGT G1-5, Amsterdam, the Netherland) equipped with a cylinder with a line density of 70 lines/cm, stylus angle of 120°, screen angle of 53°, and cell depth of 30 µm. All prints were performed in air at room temperature on corona-treated substrates. After preliminary tests, the best printing conditions were found to be a printing force of 500 N at a speed of 60 m/min. After printing, the samples were dried at 100 °C for one hour. The thickness of the dried printed layers above reported was investigated by an interferometry-based optical profilometer (Talysurf CCI HD, Taylor Hobson, Leicester, UK).

A set of ZnO NP printed samples were exposed to the vapor of 1 M acetic acid aqueous solutions for 4 h in a closed oven at 50 °C for inducing chemical bonds among the particles up to the sintering, as reported elsewhere [61].

A 40-nm-thick ZnO seed layer was also deposited on an ITO/Si (166 nm/375 µm) substrate (1.5 × 1.5 cm^2^) by ALD at 250 °C as previously reported [33]. These samples were prepared as references for the growth of ZnO NWs.

### 2.2. Growth of Nanowires

All ZnO NWs were grown under identical conditions by CBD. Samples grown on Si/ITO substrates were firstly cleaned with acetone, ethanol, and DI water in an ultrasonic bath for 5 min and dried with an N_2_ gun. For the seed layers prepared by gravure printing, the acetone and ethanol washing steps were omitted. Hexamethylenetetramine ((CH_2_)_6_N_4_, Sigma-Aldrich) and zinc nitrate hexahydrate (Zn(NO_3_)_2_.6H_2_O, Sigma-Aldrich, St. Luis, MO, USA) were dissolved in equimolar (50 mM) ratios in 150 mL of DI water at room temperature. The growth solution was stirred for 20 min at 1000 rpm and later put to rest for 40 min before decanting the clear solution into glass bottles. The substrates were fixed on cleaned glass slides with Kapton tape before being immersed face-down into the growth solution. The hydrothermal growth was carried out in an oven at 85 °C for 16 h. Later, the substrates were rinsed with DI water and dried with an N_2_ gun before being stored for surface characterization.

### 2.3. Structural Property Measurement

The printed films were morphologically characterized by atomic force microscopy (AFM) and scanning electron microscopy (SEM). AFM analysis was carried out using the Veeco Dimension Digital Instruments Nanoscope IV, New York, NY, USA.

Plainview, NY 11803 apparatus in the tapping mode configuration. For the SEM investigations of the gravure films, a field emission scanning electron microscope (FEG-SEM, Leo 1530 Gemini by Zeiss, Oberkochen, Germany) was used with an operating voltage of 7–9 kV.

Raman spectroscopy was carried out by a Renishaw InVia Reflex (Renishaw, Torino, IT) spectrometer selecting the laser wavelength of 514.5 nm (laser power of 100%) and an objective of 50× for magnification. The investigated wavelength range was 300–800 cm^−1^. For each measurement, 30 subsequent accumulations and an exposure time of 10 s were set. For each type of sample, ten measurements were taken in order to verify their homogeneity.

### 2.4. Piezoelectric and Polarity Measurement

A Bruker Dimension Icon (Santa Barbara, CA, USA) atomic force microscope instrument was used to carry out the PFM measurements on the ZnO NWs. A PtSi noncontact high-resonance frequency (NCH) tip with a high spring constant value (range values among 43–50 N/m) was used in the measurements. The PtSi-NCH tip was chosen to perform the PFM measurement as it allows a reduction of the electrostatic force involved in the piezoelectric signal [63,64]. The applied AC bias was fixed at 5 V, and the frequency was kept at 14 kHz to avoid any electrostatic contribution (typically above 50 kHz) as shown in the amplitude and phase vs. frequency plots (see Figure S1). To avoid collisions and lateral bending of the vertically grown NWs with the AFM tip, the classical contact mode PFM was not used in this study. Instead, the DataCube mode was used to measure the amplitude and phase (see the details in the Supplementary Material, including Figures S2 and S3).

## 3. Results and Discussion

In this work, gravure printing was investigated regarding the deposition of ZnO seed layers for the subsequent growth of NWs. On the base of a previous study [60], it was possible to obtain a very smooth, continuous, and homogeneous thin film of nanoparticulate ZnO seeds printed onto an ITO-coated PET substrate (see details in the Supplementary Material, including Table S1 and Figure S4). After printing, some of the printed samples were exposed to a vapor annealing sintering treatment at a very low temperature (50 °C) to investigate the effect of the seed layer morphology on NW growth.

**Figure 2:**
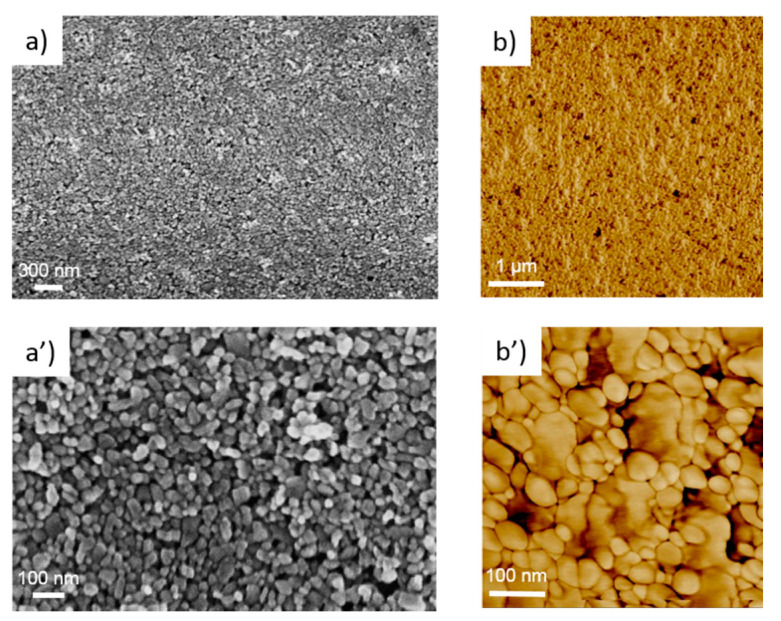
(**a**) SEM image; (**a’**) magnified SEM image; (**b**) AFM phase image; (**b’**) magnified AFM phase image of the as-printed (non-sintered) ZnO seed layer.

**Figure 3:**
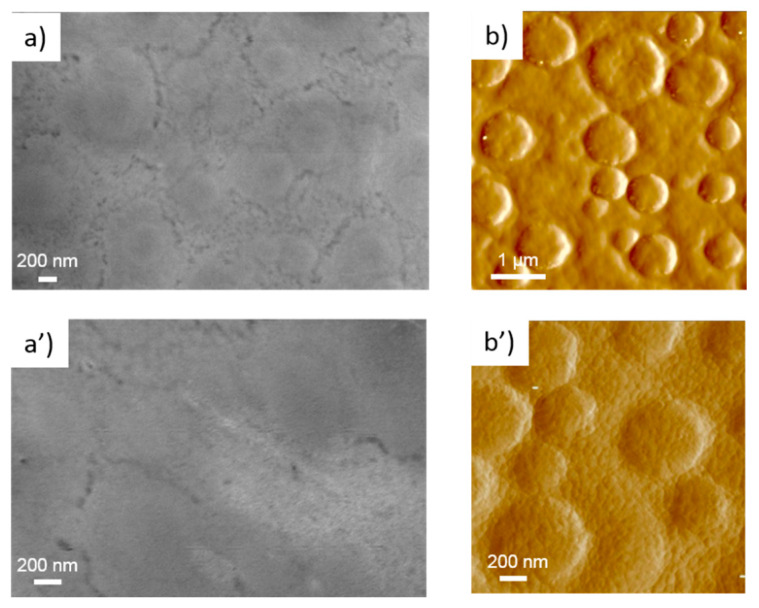
(**a**) SEM image; (**a’**) magnified SEM image; (**b**) AFM phase image; (**b’**) magnified AFM phase image of the sintered printed ZnO seed layer obtained after 4 h of treatment.

Figure 2 and Figure 3 show SEM and AFM images of the printed ZnO seed layers exposed at different annealing times of 0 and 4 h, respectively. The surface morphology changed from close-packed nanoparticulate film (as-printed) up to a dense one (sintered) over time. Indeed, thanks to a dissolution-reprecipitation mechanism during acidic vapor annealing, the chemical bonds among NPs merge particles as per a grain growth phenomenon. From these SEM images, the measured grain size in the non-sintered layers was 45 ± 12 nm. Concerning the sintered samples, although the evaluation was complex because the grain borders were not well defined, the measured values changed drastically with respect to the non-sintered layers. By the SEM analysis, the grain size ranges from 500 nm to 1000 nm with an estimated average of 547 ± 450 nm.

**Figure 4:**
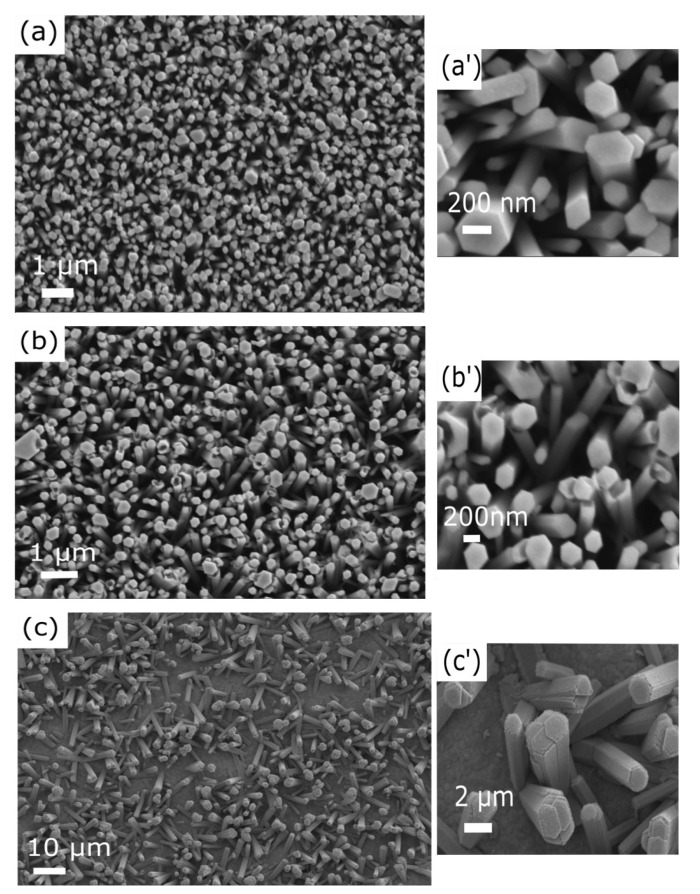
SEM images of the ZnO NWs grown on a (**a**) ZnO (ALD)/ITO/Si substrate; (**a’**) magnified image; (**b**) as-printed (non-sintered) ZnO seed layer/ITO/PET; (**b’**) magnified image; (**c**) sintered printed ZnO seed layer/ITO/PET; (**c’**) magnified image.

NWs were grown by a CBD method on both types of printed ZnO seed layers. Moreover, in order to obtain reference samples, NW growth was also performed on a Si/ITO/ZnO substrate where a ZnO seed layer was deposited by ALD. In Figure 4a–c, SEM images of the ZnO NWs on the different seed layers are shown. In Figure 4a’–c’, magnified images of the ZnO NWs are shown in a similar sequence. The ZnO NWs that were grown on the ZnO (ALD)/ITO/Si and the as-printed (not sintered) ZnO seed layer/ITO/PET substrates have average diameters of nearly 199 nm and 210 nm and standard deviations of nearly 86 nm and 84 nm, respectively. Histograms of these measurements are shown in Figure S5a,b. The ZnO NWs were closely packed and showed good vertical alignment. The obtained ZnO NWs had relatively small diameters and good alignment, which is important in piezoelectric applications [21], in particular when taking into account the following: (i) low temperature techniques of seed layer deposition; (ii) deposition on flexible substrates; and (iii) the use of non-expensive chemical methods without the requirement of a vacuum (see Table S2). In Figure 4c, it may be observed that the ZnO NWs were sparsely packed and featured random growth orientations. There was a drastic change in the dimensions of the ZnO NWs, with an average diameter of 987 nm and standard deviation of 250 nm (see Figure S5c). Figure S6a,b shows cross-sectional SEM images of ZnO NWs grown on the ITO/Si and ITO/PET substrates, respectively. It was estimated that the ZnO NWs were nearly 3.5 µm in length. It can be observed that during the growth process of ZnO NWs, some of the NWs merged. The screw-like tops of the ZnO NWs in Figure 4c indicates that their growth followed a screw dislocation nucleation mechanism along the axial direction [65].

As can be seen, the growth of NWs appeared to be significantly affected by the morphology of the seed layer, as also stated in the literature [66,67,68]; however, the relationships between the seed layer characteristics and NW growth have not been clearly established as of yet. In particular, the seed layer thickness, orientation, and grain size have key roles on the vertical growth of ZnO NWs. As known, ZnO preferentially crystallizes in a hexagonal polar wurtzite structure [69]. The ZnO NW growth direction by CBD occurs predominantly along the polar direction of the seed layer, which provides nucleation sites [66,67].

A possible explanation for the observed results may be proposed on a thermodynamic basis, considering the processes driven by the minimization of the overall system energy. In particular, the seed layer energy governs the nucleation process and subsequently determines the density of ZnO NWs.

In general, the energy of a particle is a function of the size, shape, stress, and external environment [70]. In a NP, a significant number of atoms are localized at the surface [69]. The additional energetic term due to the high-energy under-coordinated atoms at the surface is described as the surface free energy [71,72] and becomes increasingly larger as particle size decreases. In addition, several surface defects [70,73,74] may be present, especially when the particle size is very small [75], resulting in a further increase in the particle free energy [76]. As such, due to their high energy, NPs typically show a higher reactivity compared to bulk or micrometric particles [77,78]. As a result, a highly energetic as-printed nanoparticulate seed layer can be especially chemically reactive for ZnO NW nucleation. In addition, a NP seed alignment mechanism may be present during the CBD, furthering contributing to the high NW density. As shown in Figure 2, the as-printed seed layer was composed of near-spherical NPs having a wurtzite structure, as demonstrated by Raman spectroscopy (see Figure S7 and Table S3). As with most thin polycrystalline films, nanoparticulate printed layers are not textured [66,79], namely, a random nanocrystal orientation is expected. In fact, in the gravure process, there is no particular force that is able to induce a preferential nanoparticle alignment. The final NP arrangement is determined by the strongly attractive lateral capillary forces among particles during solvent evaporation [60,80]. On the other hand, the surface energy distribution of wurtzite-type ZnO NPs is anisotropic and varies between crystallographic orientations [69,81]. Since the NPs are roughly spherical, a large variety of crystallographic planes are presumably exposed to the CBD process [82]. Among these, polar surfaces are extremely chemically reactive [83,84] and have been found to be dominant surface nucleation sites for the growth of ZnO NWs in a solution [66]. To this regard, it has been observed that a high seed layer texture along the polar axis is necessary to obtain a high NW density [67,85,86]. Furthermore, high-density and well-orientated NWs (see Figure 4b) may support a heat-triggered reorientation mechanism for random NPs during the early stages of the CBD [87]. This is possible as the NPs are stuck together by weak physical forces, where they can consequently easily rotate due to the strong electrostatic interactions between charged species (ions, particles, and embryonic dipoles) in the chemical bath, thus reducing the high-energy polar surfaces of wurtzite by nucleation. For these reasons, the as-printed nanoparticulate seed layer demonstrated here can offer a very high number of high-energy nucleation sites (potentially each seed can become a NW, unless steric hindrance interferes). This factor is responsible for the high NW density along the polar c-axis of the hexagonal wurzite crystal (see Figure 4b). Moreover, due to the small sizes of the seeds, the NW diameters were very small, which is typically a challenging result to achieve with most wet chemical methods [88]. Qualitatively, from the SEM images (Figure 4a,b), the NWs grown on the as-printed (non-sintered) layer are comparable in terms of diameter and verticality to the ones grown on the ALD deposited layers.

On the contrary, in the case of a sintered printed sample, far more nucleation sites at lower energy are exposed to the CBD process, resulting in a poor final NW density (see Figure 4c). In fact, sintering is an interface elimination process that decreases the surface area, this creating bonds between contacting particles [89,90]. The total free energy of the system is reduced by replacing the high thermodynamic chemical potential of convex free surfaces of the NPs into lower energy grain boundaries through NP merging. Furthermore, during the densification, the grain boundaries migrate for further reduction of the total free energy, thus resulting in a grain growth phenomenon [91]. Since the grain boundary energy is anisotropic, a lower energy grain boundary tends to extend the most [91,92,93,94]. As such, the surfaces of the sintered seeds are mainly dominated by electrically neutral planes [95], which are sites that are not suitable for NW nucleation. Furthermore, the grown grains become trapped by the chemical bonds between grain boundaries. As a result, only some grains with a polar surface available for growth can support nanowires. Such results appear consistent with the observations regarding the decrease in NW density as the seed layer grain size increases [96]. Nevertheless, a chemical sintering post-treatment could be investigated in future to induce potential bonding among seeds and between the seeds and substrate, as well as for tuning the nanowire characteristics.

In some recently published articles, it can be found that surfactants like trisodium citrate, ethylenediamine, poly(ethylene glycol), cetyltrimethylammonium bromide, and sodium dodecyl sulphate are used as surfactants, whose role is to change the surface free energy of the different ZnO crystal facets [97,98]; however, in this work, the role played by the surfactants was complemented by the presence of the seed layer on the substrate.

It is also necessary to discuss the role played by the HMTA in the formation of the ZnO NWs via a CBD process. Although the exact role of HMTA is still under debate, it is believed that the reagent plays multiple roles in the formation of ZnO nanocrystals. HMTA is hydrolyzed upon heating into formaldehyde and ammonia. The latter is further protonated in water to produce OH^‒^ ions, thus increasing the solution pH. It was found that the rate of decomposition of HMTA is dependent on the concentration of protons in the solution and is independent to the precipitation of ZnO [99,100]. Consequently, it was argued that HMTA fulfills the role of a pH buffer since the rate of hydrolysis is decreases with an increased pH [101].

In more recent works, it has been shown that the crystal morphology is also strongly influenced by the chemical precursor concentration. A better crystal stoichiometry data have been obtained in the films deposited with larger amounts of HMTA in solution, thus reducing the number of oxygen vacancies and interstitial zinc in the lattice [102].

Additionally, it is believed that HMTA inhibits the growth of nonpolar m-plane sidewalls, thus favoring an anisotropic growth along the c-axis [103].

### Piezoelectric Characterization

**Figure 5:**
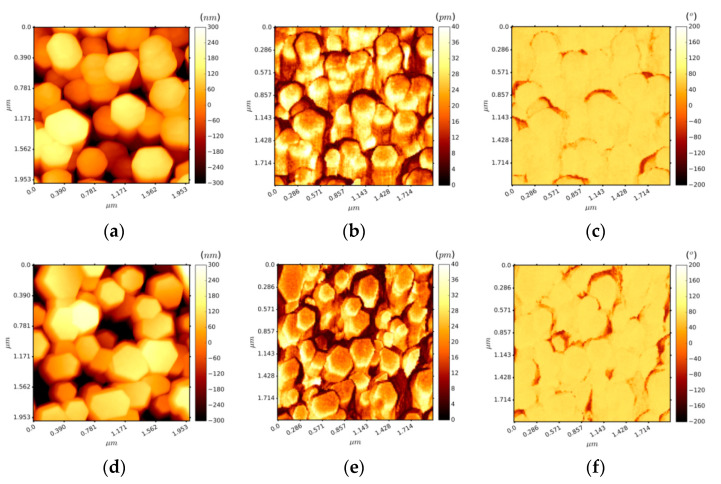
PFM images of the (**a**,**b**) topography, (**c**,**d**) amplitude, and (**e**,**f**) phase of the ZnO NW growth on ZnO (ALD)/ITO/Si (images lined up above) and as-printed (not sintered) ZnO seed layer/ITO/PET substrates (images lined up below).

To explore the effectiveness of the gravure printing method compared to the ALD process for growing ZnO seed layers on the flexible substrates, we carried out PFM measurements on the ZnO (ALD)/ITO/Si and as-printed (not sintered) ZnO seed layer/ITO/PET samples. The PFM is a widely used tool to study the polarity and the piezo-response properties of piezoelectric thin films and NWs. In this work, the PFM was carried out using the DataCube mode to study the polarities on the top surface of ZnO NWs by recording the phase signals. The $d_{33}$ piezoelectric coefficients were computed by analysing the piezoelectric amplitudes as presented in Figure 5a–f.

**Figure 6:**
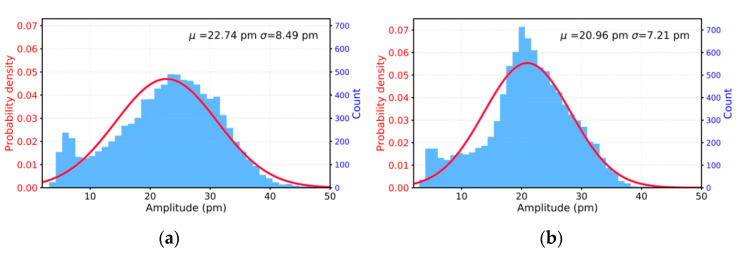
Piezoelectric amplitude histograms of ZnO NW growth on (**a**) ZnO (ALD)/ITO/Si and (**b**) as-printed (not sintered) ZnO seed layer/ITO/PET substrates. The red curve is the Gaussian fitting function.

Figure 5a,b show the topography in an area of 2 × 2 µm^2^ in a top-view of ZnO NWs grown on ITO/Si and ITO/PET structures. At first view, we can see good uniformity and an excellent hexagonal shape for the ZnO NWs as per the SEM results (Figure 4). Besides, they have the same correlation regarding their diameter (between 53 and 210 nm) and roughness parameters at the top end of ZnO (e.g., root mean square (Rq) values are approximately 120 nm). Figure 5c,d show the PFM amplitudes for both samples when an AC drive voltage of 5 V was applied. The mean amplitude values (µ) were very similar (22.74 ± 8.49 pm and 20.96 ± 7.21 pm, as shown in Figure 6, for ITO/Si and ITO/PET, respectively) according to the Gaussian fitting function for piezoelectric amplitude distribution at the top surface of ZnO NWs. Based on these results, we estimated the effective piezoelectric coefficients $\left(d_{33}^{eff}\right)$ to be 4.6 and 4.1 pm/V for the ZnO NWs grown on ITO/Si and ITO/PET, respectively. It is important to highlight that both samples presented almost the same $d_{33}^{eff}$ values and they were inside the range of the previously reported experimental results of 2–12 pm/V [104,105,106,107,108].

On the other hand, the PFM measurements also provided results concerning the piezoelectric phase as depicted in Figure 5e,f. Both results show a single-phase value of around 75° (see Figure S3) throughout the top surface of ZnO NWs, thus demonstrating the Zn surface polarity of the crystal structure [64]. Obtaining a homogeneous surface polarity is very important at a device level, i.e., where NWs are typically integrated and placed between electrodes [33]. If the NWs had shown negative and positive phase values in their top surface, the induced negative and positive electrical charges would have cancelled out, thus reducing the overall piezoelectric response at the device level.

## 4. Conclusions

In summary, our experimental results show that gravure printing is a promising low-temperature ZnO seeding method, especially from the perspective of fabricating flexible piezoelectric devices. Study of the printed seed layer morphology revealed that the nanoparticulate allowed a better growth of vertical ZnO NWs by CBD with respect to the sintered one because the high-energy NPs provided a higher number of nucleation sites. To explain the observed results, a possible reorientation mechanism of the ZnO nanoparticle seeds was also proposed. PFM measurements of the NWs revealed (i) a Zn-phase polarity with a very good homogeneity, which is essential to obtaining globally optimal piezoelectric performance when integrated in devices, and (ii) an estimated piezoelectric coefficient close to 4 pm/V, which is comparable to the NWs grown on Si/ITO substrate.

The mobility and flexibility of the whole structure, provided by the use of flexible ZnO NWs, is expected to have a good impact on the device’s mechanical performance and durability.

This work shows the way towards the fabrication of flexible piezoelectric transducers based on ZnO nanowires. Fabrication is completely carried out by solution processes at a low temperature.

## Data Availability

The data are available upon request from the corresponding authors.

## Supplementary Materials

The following are available online at https://www.mdpi.com/article/10.3390/nano11061430/s1, Figure S1: sweep of drive frequency value of AC bias signal for PFM amplitude and phase values in different ranges of frequency; Figure S2: single frame of (a) amplitude and (b) phase measurement and average value of 20 frames of (c) amplitude and (d) phase; Figure S3: piezoelectric phase distribution value of ZnO NWs grown on (a) ZnO (ALD)/ITO/Si and (b) as-printed (non-sintered) ZnO seed layer/ITO/PET substrates. The red curve is the fitting curve using Gaussian mixture models, Figure S4: height distribution of the top surface of (a) ITO over PET, (b) ITO over Si, and (c) ZnO seed layer (deposited by ALD) over ITO/Si to extract the roughness parameters by AFM; Figure S5: diameter distribution of ZnO NWs grown on (a) ZnO (ALD)/ITO/Si and (b) as-printed (non-sintered) ZnO seed layer/ITO/PET substrates. The red curve is the Gaussian fitting function; Figure S6: SEM image of the cross-section of the ZnO NWs grown on (a) ITO/Si substrate and (b) ITO/PET substrate; Figure S7: Raman spectra of ZnO seed layer films deposited onto aluminium by gravure printing measured before and after 4 h of sintering; Table S1: arithmetical mean deviation values (Ra) and root squared values (Rq) measured for the different substrates and manufactured ZnO seed layers; Table S2: comparison table of ZnO NWs grown by chemical bath deposition (CBD) from recent articles; Table S3: assignments for the Raman bands marked in Figure S2 to specific vibrations for ZnO structures.
