# Supplementary material for: Low-Temperature Growth of ZnO Nanowires from Gravure-Printed ZnO Nanoparticle Seed Layers for Flexible Piezoelectric Devices
Source: Nanomaterials (Basel). 2021 May 28;11(6):1430. doi: 10.3390/nano11061430 (PMC8226623; doi:10.3390/nano11061430)
Supplement: Supplementary file 1 [file nanomaterials-11-01430-s001.zip › nanomaterials-1208638-supplementary.pdf]

nanomaterials

Supplementary material

Low-Temperature Growth of ZnO Nanowires from Gravure-Printed ZnO Nanoparticle Seed Layers for Flexible Piezoelectric Devices

Andrés Jenaro Lopez Garcia [1,†], Giuliano Sico [2,†], Maria Montanino [2,†], Viktor Defoor [1], Manojit Pusty [1], Xavier Mescot [1], Fausta Loffredo [2], Fulvia Villani [2], Giuseppe Nenna [2,*] and Gustavo Ardila [1,*]

[1] University Grenoble Alpes, University Savoie Mont Blanc, CNRS, Grenoble INP, IMEP-LaHC, F-38000 Grenoble, France; andres-jenaro.lopez-garcia@grenoble-inp.fr (A.J.L.G.); viktor.defoor@grenoble-inp.fr (V.D.); manojit.pusty@grenoble-inp.fr (M.P.); xavier.mescot@grenoble-inp.fr (X.M.)
[2] ENEA, Italian National Agency for New Technologies, Energy and Sustainable Economic Development, Portici Research Centre, P.le E. Fermi 1, Portici, I-80055 Naples, Italy; giuliano.sico@enea.it (G.S.); maria.montanino@enea.it (M.M.); fausta.loffredo@enea.it (F.L.), fulvia.villani@enea.it (F.V.)
* Correspondence: giuseppe.nenna@enea.it (G.N.); ardilarg@minatec.grenoble-inp.fr (G.A.); Tel.: +33-456-529-532 (G.A.)
† These authors contributed equally to the work.

PFM Measurements

During PFM measurement, the ITO seed layer was directly connected to the chuck of the AFM system using copper tape. The tip was put in contact with the ZnO NWs sample, and an alternating current (AC) bias was applied to the tip during the scanning process. The frequency was kept at 14 kHz to avoid any electrostatic contribution (typically above 50 kHz) in a range of frequency in which amplitude and phase are more stable (see Figure S1). Accordingly, the top of ZnO NWs undergoes a deformation owing to the converse piezoelectric effect. This results in the deflection of the cantilever tip which is detected by a photodiode and subsequently analyzed by a lock-in amplifier system. Overall, two quantities were measured during PFM characterization: the amplitude of the deformation and the phase. The former is the result of an expansion of the analyzed domain when both vectors (i.e., electric field and polarization) are aligned, thus deflecting the cantilever tip, whereas the latter is due to the alignment angle between the electric field of the applied alternating signal, and the polarization vector of the piezoelectric domains present within the sample.

Normally, the PFM measurement operates in continuous mechanical contact between the tip and sample during the scanning process, but some difficulties were found using this technique. Multiple collisions between the tip and the ZnO NWs were produced since the sample has a high roughness because of the free space that exists between NWs. To overcome this difficulty, the DataCube mode was used to measure the amplitude and phase. The measurements were carried out over large areas (around 2 µm × 2 µm) on top of the surfaces of the ZnO NW arrays. Before measuring with the DataCube mode, the topography of the sample was acquired using a tapping mode technique to determine the surface roughness parameters, which were then used as input parameter for the Z ramp size in the DataCube mode. In the DataCube mode, the tip is placed locally over the top surface of ZnO NW, keeping its position under control by adjusting two parameters, namely, the Z ramp size and the force applied (controlled by the tip deflection parameters) for each scanned pixel. In addition, all PFM measurements were made with a constant tip deflection of 20 nm. Simultaneously, the amplitude and phase quantities were measured during a dwell time (or hold time) of 80 ms. Furthermore, during

this time, 208 frames of a 2 µm × 2 µm surface area were recorded to analyze both amplitude and phase. To improve the measurement accuracy and image quality, 20

frames of the amplitude and phase values were randomly taken from the hold time range of recorded the DataCube file using the Nanoscope software (Bruker Corporation, Billerica (Massachusetts), United States) and then averages of the 20 frames were compiled into a single image for both amplitude and phase using the Python software (Python Software Foundation, Wilmington (Delaware), United States) program (see Figure S2).

Finally, Figure S3 shows the piezoelectric phase distribution value for the Si/ITO and PET/ITO substrates.

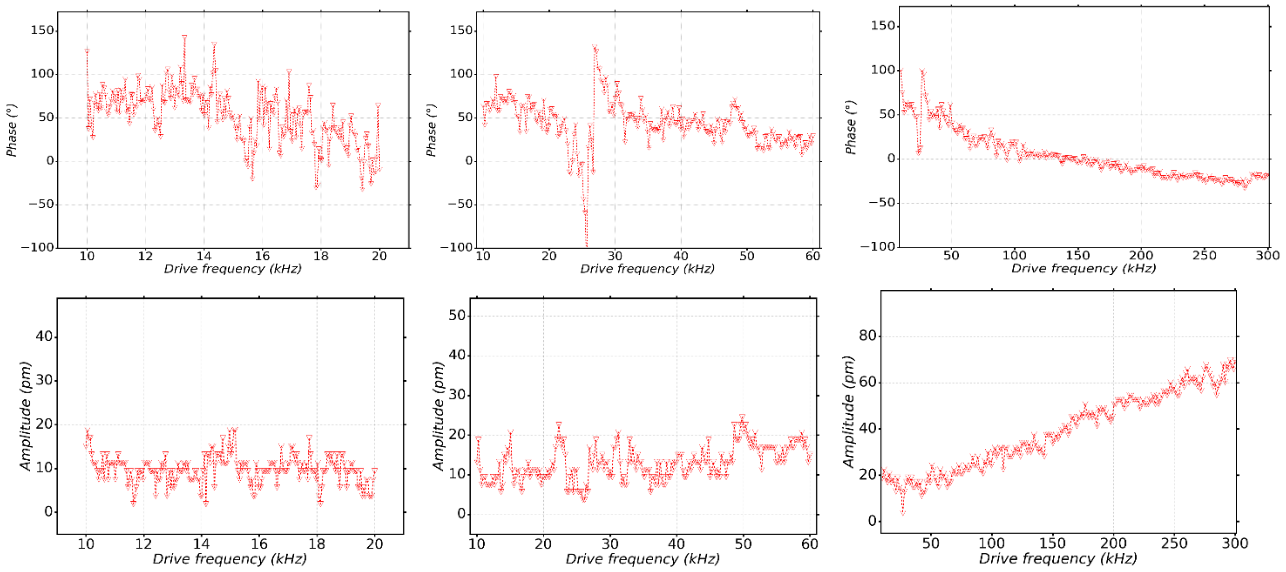

Figure S1. Sweep of the drive frequency value for the AC bias signal for the PFM amplitude and phase values in different frequency ranges.

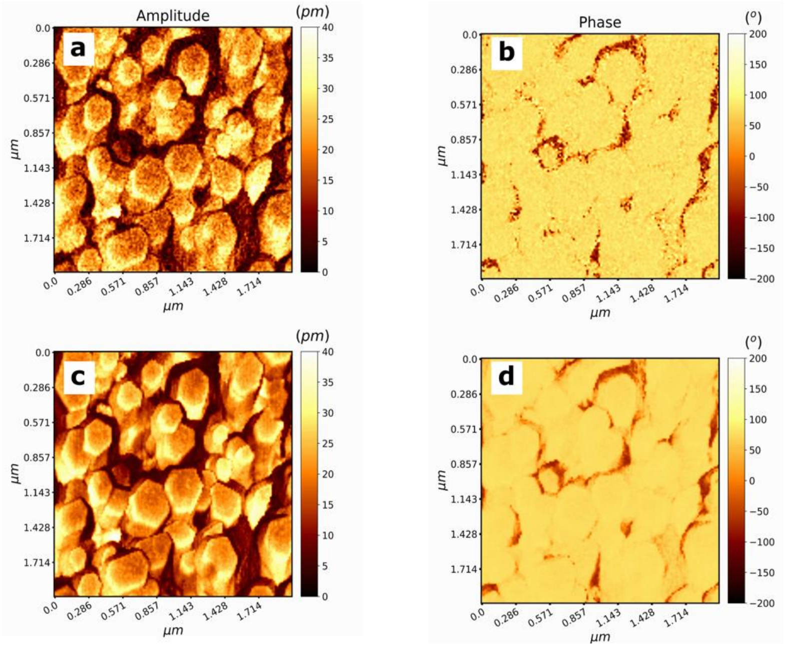

Figure S2. A single frame of (**a**) amplitude and (**b**) phase measurement and average values of 20 frames of (**c**) amplitude and (**d**) phase.

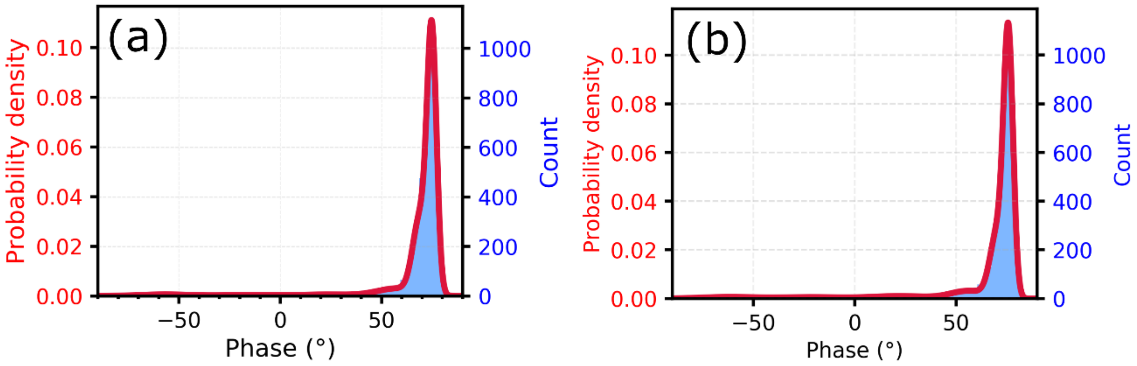

Figure S3. Piezoelectric phase distribution values of ZnO NWs grown on (**a**) ZnO (ALD)/ITO/Si and (**b**) the as-printed (not sintered) ZnO seed layer/ITO/PET substrates. The red curve is the fitting curve using Gaussian mixture models.

Table S1 reports the arithmetical mean deviation values (Ra) and root squared values (Rq) measured by AFM for the different substrates (Figure S4b and Figure S4c) and manufactured ZnO seed layers.

Thin Film Characterization

Table S1. Arithmetical mean deviation values (Ra) and root squared values (Rq) measured for the different substrates and manufactured ZnO seed layers.

Samples	Ra (nm)	Rq (nm)
ITO/Si	1.70	2.14
ZnO seed layer (ALD) /ITO/Si	1.04	1.35
ITO/PET	1.50	2.11
Not sintered ZnO seed layer (gravure) /ITO/PET	1.49	1.97
4h sintered ZnO seed layer (gravure) /ITO/PET	10.9	13.5

The sheet resistance of the ITO (0.18µm)/Si was measured by a 4-point probe method, giving a value of 34 Ω/Sq, while for the ITO (0.13 µm)/PET the sheet resistance was 58 Ω/Sq.

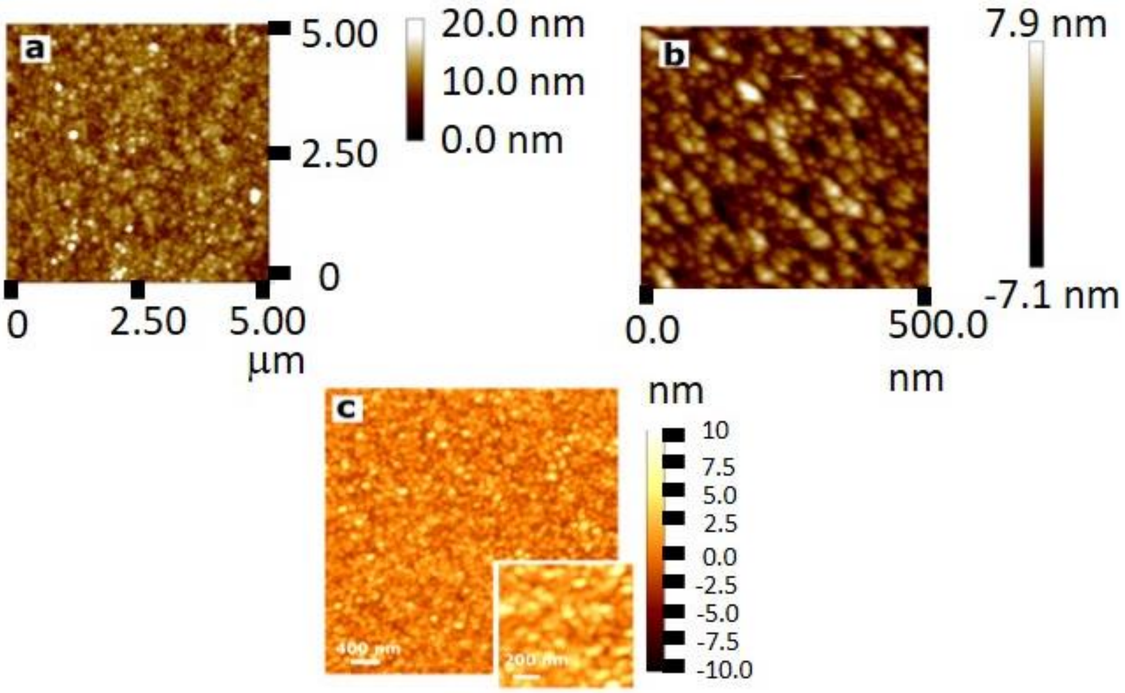

Figure S4. Height distribution of the top surface of (**a**) ITO over PET, (**b**) ITO over Si, and (**c**) the ZnO seed layer (deposited by ALD) over ITO/Si to extract the roughness parameters by AFM.

ZnO NW Diameter Distribution

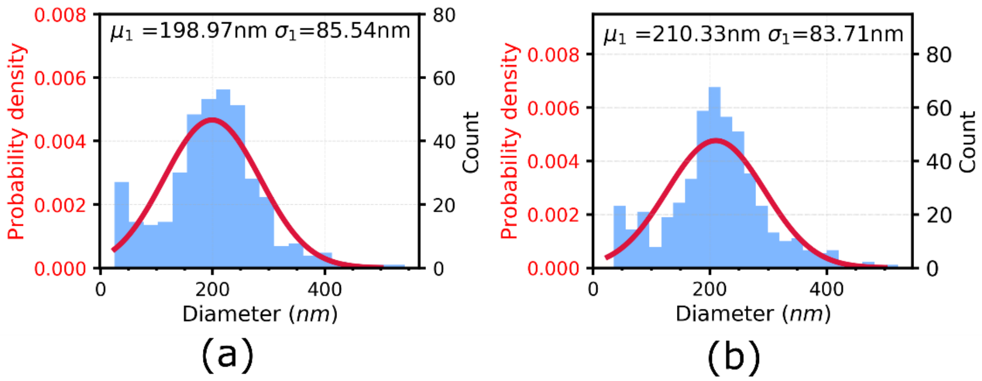

Figure S5. Diameter distribution of ZnO NWs grown on (**a**) ZnO (ALD)/ITO/Si and (**b**) the as-printed (not sintered) ZnO seed layer/ITO/PET substrates. The red curve is the Gaussian fitting function.

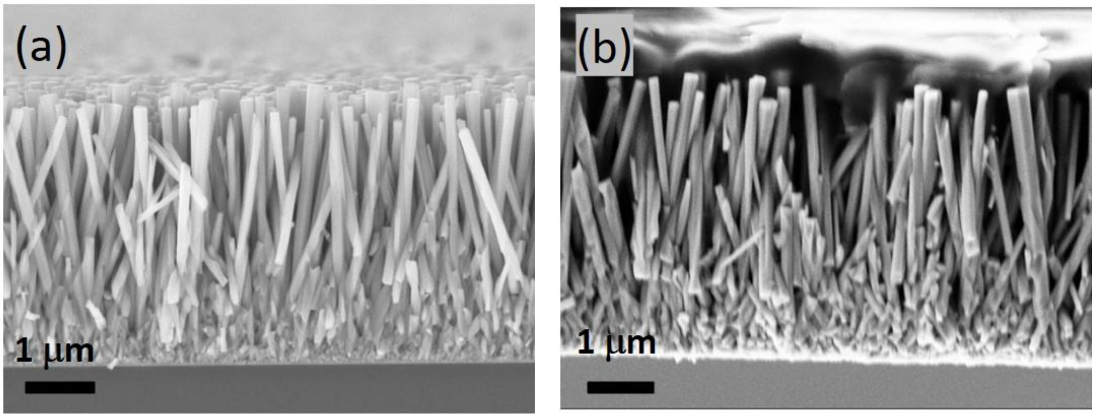

Figure S6. SEM image of the cross section of the ZnO NWs grown on the (**a**) ITO/SI and (**b**) ITO/PET substrates.

Table S2. Comparison of ZnO NWs grown by chemical bath deposition (CBD) from recent articles.

Diameter (nm)	Length (μm)	NW Orientation	Substrate	Flexible	Seed Layer Material	Seed Layer Deposition Technique	Seed Layer/NW Growth Solution Temperature	Ref.
266	1.2	Poor	ITO	–	–	–	– / 85 °C	[1]
580	6.7	Good	Si	No	Au	Vacuum evaporation	– / 85 °C	[2]
50	0.74	Good	Si	No	Zinc acetate solution	Spin coating, annealing	Room temperature (RT) deposition, then annealing at 400 °C/83 °C	[3]
180	3		Glass	No	ZnO	Radio frequency (RF) sputtering	Seed layer annealed at 400 °C/–	[4]
–	–	Good	Si	No	ZnO nanoparticle	Sol-gel	RT deposition, then annealing at 300 °C/95 °C	[5]
150	2	Good	Si	No	ZnO	E-beam evaporation	–/ 90 °C	[6]
210	2	Poor	Glass coverslip	No	MnOOH	$KMnO_4$	Soaking in $KMnO_4$ at 90 °C/ 85 °C	[7]
50	0.3	Good	ZnO/ITO/PET	Yes	ZnO	RF sputtering	Deposition on PET at 120 °C/ 75 °C	[8]
100-250	2	Good	Si as a temporary substrate	Yes	Au/Ti	Thermal evaporation	RT deposition then annealing at 300 °C/–	[9]
500	5	Good	PES	Yes	ZnO	Sol-gel	RT deposition then annealing at 150 °C/90 °C	[10]
70	2	Poor	PI	Yes	ZnO	Roll-to-roll printing	RT deposition then annealing at 350 °C/92 °C	[11]
74	15	Poor	Copper PCB	Yes	MnOOH	Soaking in $KMnO_4$	Soaking in $KMnO_4$ at 90 °C/ 85 °C	[12]
210	3.5	Good	ITO/PET	Yes	ZnO	Gravure printing	RT printing then annealing at 100 °C/85 °C	Present Work

Raman Spectroscopy

Raman spectroscopy was used to study the crystal structure of the ZnO films deposited by gravure printing onto aluminium. The Raman spectra of ZnO measured before and after sintering treatment (4h) in the region from 300 to 700cm^{-1}, are reported in Figure S7. For all the samples two principal phonon bands, centered at 438 and 578 cm^{-1} are well

visible. According to the literature [13–18] they can be associated to phonon modes in ZnO structures as shown in Table S3. In particular, the intensity of the strong band centered at 438cm^{-1} is, associated to a non-polar E_2^{high} (O) phonon mode of the oxygen anions present in wurtzite hexagonal phase of ZnO. [16]

Table S3. Assignments for the Raman bands marked in Figure S7 to specific vibrations of ZnO structures.

Wavenumber (cm^{-1})	Vibration mode	Ref.
438	E2 (high)	[13],[15],[17],[18]
578	LO quasimode with mixed A1 and E1 symmetry	[19]

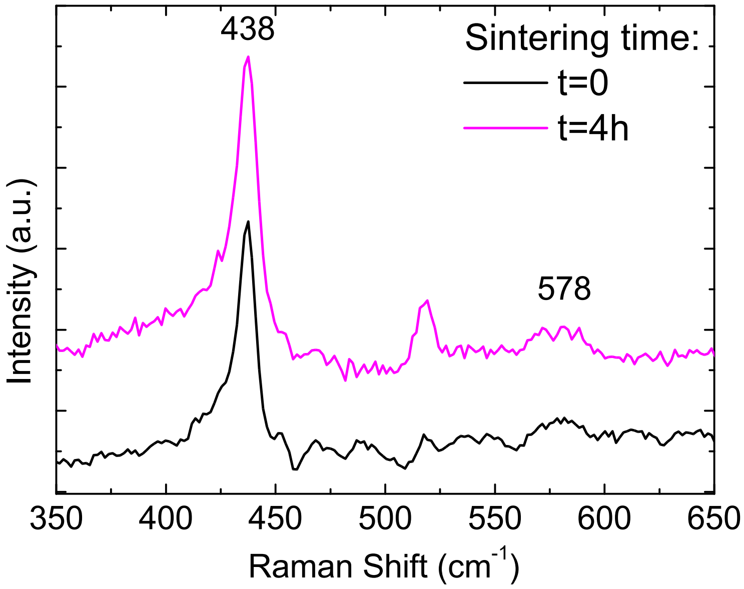

Figure S7. Raman spectra of ZnO seed layer films deposited onto aluminium by gravure printing as measured before and after 4 h of sintering.

References

1. Abdelfatah, M.; Salah, H.Y.; EL-Henawey, M.I.; Oraby, A.H.; El-Shaer, A.; Ismail, W. Insight into Co concentrations effect on the structural, optical, and photoelectrochemical properties of ZnO rod arrays for optoelectronic applications. *J. Alloys Compd.* **2021**, *873*, 159875, doi:10.1016/j.jallcom.2021.159875.
2. Lausecker, C.; Salem, B.; Baillin, X.; Chaix-Pluchery, O.; Roussel, H.; Labau, S.; Pelissier, B.; Appert, E.; Consonni, V. Chemical Bath Deposition of ZnO Nanowires Using Copper Nitrate as an Additive for Compensating Doping. *Inorg. Chem.* **2021**, *60*, 1612–1623, doi:10.1021/acs.inorgchem.0c03086.
3. Huang, Y.C.; Zhou, J.; Nomenyo, K.; Ionescu, R.E.; Gokarna, A.; Lerondel, G. Facile, wafer-scale compatible growth of ZnO nanowires: Via chemical bath deposition: Assessment of zinc ion contribution and other limiting factors. *Nanoscale Adv.* **2020**, *2*, 5288–5295, doi:10.1039/d0na00434k.
4. Abdulrahman, A.F.; Ahmed, S.M.; Barzinjy, A.A.; Hamad, S.M.; Ahmed, N.M.; Almessiere, M.A. Fabrication and characterization of high-quality uv photodetectors based zno nanorods using traditional and modified chemical bath deposition methods. *Nanomaterials* **2021**, *11*, 1–26, doi:10.3390/nano11030677.
5. Saha, R.; Karmakar, A.; Chattopadhyay, S. Enhanced self-powered ultraviolet photoresponse of ZnO nanowires/p-Si heterojunction by selective in-situ Ga doping. *Opt. Mater. (Amst).* **2020**, *105*, 109928, doi:10.1016/j.optmat.2020.109928.
6. Geng, Y.; Jeronimo, K.; Mahzan, M.A.B.C.; Lomax, P.; Mastropaolo, E.; Cheung, R. Comparison of ZnO Nanowires

Grown on E-beam Evaporated Ag and ZnO seed layers. *Nanoscale Adv.* **2020**. https://doi.org/10.1039/C9NA00553F.

7. Errico, V.; Arrabito, G.; Fornetti, E.; Fuoco, C.; Testa, S.; Saggio, G.; Rufini, S.; Cannata, S.; Desideri, A.; Falconi, C.; et al. High-Density ZnO Nanowires as a Reversible Myogenic-Differentiation Switch. *ACS Appl. Mater. Interfaces* **2018**, *10*, 14097–14107, doi:10.1021/acsami.7b19758.
8. Lee, C.Y.; Li, S.Y.; Lin, P.; Tseng, T.Y. ZnO nanowires hydrothermally grown on PET polymer substrates and their characteristics. *J. Nanosci. Nanotechnol.* **2005**, *5*, 1088–1094. doi: 10.1166/jnn.2005.164.
9. Lin, H.-I.; Wuu, D.-S.; Shen, K.-C.; Horng, R.-H. Fabrication of an ultra-flexible ZnO nanogenerator for harvesting energy from respiration. *ECS J. Solid State Sci. Technol.* **2013**, *2*, P400.
10. Kim, H.G.; Kim, E.H.; Kim, S.S. Growth of ZnO Nanorods on ITO Film for Piezoelectric Nanogenerators. *Materials (Basel).* **2021**, *14*, 1461-1471. https://doi.org/10.3390/ma14061461
11. Park, J.; Lee, J.; Noh, Y.; Shin, K.-H.; Lee, D. Flexible ultraviolet photodetectors with ZnO nanowire networks fabricated by large area controlled roll-to-roll processing. *J. Mater. Chem. C* **2016**, *4*, 7948–7958. https://doi.org/10.1039/C6TC02371A.
12. Arrabito, G.; Errico, V.; Zhang, Z.; Han, W.; Falconi, C. Nanotransducers on printed circuit boards by rational design of high-density, long, thin and untapered ZnO nanowires. *Nano Energy* **2018**, *46*, 54–62, doi:10.1016/j.nanoen.2018.01.029.
13. Ojha, A.K.; Srivastava, M.; Kumar, S.; Hassanein, R.; Singh, J.; Singh, M.K.; Materny, A. Influence of crystal size on the electron–phonon coupling in ZnO nanocrystals investigated by Raman spectroscopy. *Vib. Spectrosc.* **2014**, *72*, 90–96. https://doi.org/10.1016/j.vibspec.2014.02.013
14. Lu, J.G.; Kawaharamura, T.; Nishinaka, H.; Kamada, Y.; Ohshima, T.; Fujita, S. ZnO-based thin films synthesized by atmospheric pressure mist chemical vapor deposition. *J. Cryst. Growth* **2007**, *299*, 1–10. https://doi.org/10.1016/j.jcrysgro.2006.10.251.
15. Bendall, J.S.; Visimberga, G.; Szachowicz, M.; Plank, N.O. V; Romanov, S.; Sotomayor-Torres, C.M.; Welland, M.E. An investigation into the growth conditions and defect states of laminar ZnO nanostructures. *J. Mater. Chem.* **2008**, *18*, 5259–5266. https://doi.org/10.1039/B812867G.
16. Bedia, A.; Bedia, F.Z.; Aillerie, M.; Maloufi, N.; Hamady, S.O.S.; Perroud, O.; Benyoucef, B. Optical, electrical and structural properties of nano-pyramidal ZnO films grown on glass substrate by spray pyrolysis technique. *Opt. Mater. (Amst).* **2014**, *36*, 1123–1130. https://doi.org/10.1016/j.optmat.2014.02.012
17. Dobrozhan, O.; Opanasyuk, A.; Kolesnyk, M.; Demydenko, M.; Cheong, H. Substructural investigations, Raman, and FTIR spectroscopies of nanocrystalline ZnO films deposited by pulsed spray pyrolysis. *Phys. status solidi* **2015**, *212*, 2915–2921. https://doi.org/10.1002/pssa.201532324
18. Šćepanović, M.; Grujić-Brojčin, M.; Vojisavljević, K.; Bernik, S.; Srećković, T. Raman study of structural disorder in ZnO nanopowders. *J. Raman Spectrosc.* **2010**, *41*, 914–921. https://doi.org/10.1002/jrs.2546
19. Bergman, L.; Chen, X.-B.; Huso, J.; Morrison, J.L.; Hoeck, H. Raman scattering of polar modes of ZnO crystallites. *J. Appl. Phys.* **2005**, *98*, 93507. https://doi.org/10.1063/1.2126784
